# Analysis of ddRAD-seq data provides new insights into the genomic structure and patterns of diversity in Italian donkey populations

**DOI:** 10.1093/jas/skae165

**Published:** 2024-06-14

**Authors:** Andrea Criscione, Giorgio Chessari, Alberto Cesarani, Michela Ablondi, Vittoria Asti, Daniele Bigi, Salvatore Bordonaro, Roberta Ciampolini, Claudio Cipolat-Gotet, Michele Congiu, Pasquale De Palo, Vincenzo Landi, Nicolò Pietro Paolo Macciotta, Donato Matassino, Baldassare Portolano, Silvia Riggio, Alberto Sabbioni, Maria Teresa Sardina, Gabriele Senczuk, Serena Tumino, Matteo Vasini, Elena Ciani, Salvatore Mastrangelo

**Affiliations:** Dipartimento di Agricoltura, Alimentazione e Ambiente, University of Catania, Catania, Italy; Dipartimento di Agricoltura, Alimentazione e Ambiente, University of Catania, Catania, Italy; Department of Animal Sciences, Georg-August-University Göttingen, Göttingen, Germany; Dipartimento di Agraria, University of Sassari, Sassari, Italy; Department of Animal and Dairy Science, University of Georgia, Athens, USA; Dipartimento di Scienze Medico-Veterinarie, University of Parma, Parma, Italy; Dipartimento di Scienze Medico-Veterinarie, University of Parma, Parma, Italy; Dipartimento di Scienze e Tecnologie Agro-Alimentari, University of Bologna, Bologna, Italy; Dipartimento di Agricoltura, Alimentazione e Ambiente, University of Catania, Catania, Italy; Dipartimento di Scienze Veterinarie, University of Pisa, Pisa, Italy; Dipartimento di Scienze Medico-Veterinarie, University of Parma, Parma, Italy; Dipartimento di Agraria, University of Sassari, Sassari, Italy; Dipartimento di Medicina Veterinaria, University of Bari Aldo Moro, Valenzano, Italy; Dipartimento di Medicina Veterinaria, University of Bari Aldo Moro, Valenzano, Italy; Dipartimento di Agraria, University of Sassari, Sassari, Italy; Consorzio per la Sperimentazione, Divulgazione e Applicazione di Biotecniche Innovative, Benevento, Italy; Dipartimento di Scienze Agrarie, Alimentari e Forestali, University of Palermo, Palermo, Italy; Dipartimento di Scienze Agrarie, Alimentari e Forestali, University of Palermo, Palermo, Italy; Dipartimento di Scienze Medico-Veterinarie, University of Parma, Parma, Italy; Dipartimento di Scienze Agrarie, Alimentari e Forestali, University of Palermo, Palermo, Italy; Dipartimento di Agricoltura, Ambiente e Alimenti, University of Molise, Campobasso, Italy; Dipartimento di Agricoltura, Alimentazione e Ambiente, University of Catania, Catania, Italy; Associazione Nazionale Allevatori delle Razze Equine ed Asinine Italiane, ANAREAI, Roma, Italy; Dipartimento di Bioscienze, Biotecnologie e Biofarmaceutica, University of Bari Aldo Moro, Bari, Italy; Dipartimento di Scienze Agrarie, Alimentari e Forestali, University of Palermo, Palermo, Italy

**Keywords:** donkey, SNP markers, genetic diversity, adaptation, conservation

## Abstract

With more than 150 recognized breeds, donkeys assume relevant economic importance, especially in developing countries. Even if the estimated number of heads worldwide is 53M, this species received less attention than other livestock species. Italy has traditionally been considered one of the cradles of European donkey breeding, and despite a considerable loss of biodiversity, today still counts nine autochthonous populations. A total of 220 animals belonging to nine different populations were genotyped using the double-digest restriction site associated DNA (**ddRAD**) sequencing to investigate the pattern of diversity using a multi-technique approach. A total of 418,602,730 reads were generated and successfully demultiplexed to obtain a medium-density SNP genotypes panel with about 27K markers. The diversity indices showed moderate levels of variability. The genetic distances and relationships, largely agree with the breeding history of the donkey populations under investigation. The results highlighted the separation of populations based on their genetic origin or geographical proximity between breeding areas, showed low to moderate levels of admixture, and indicated a clear genetic difference in some cases. For some breeds, the results also validate the success of proper management conservation plans. Identified runs of homozygosity islands, mapped within genomic regions related to immune response and local adaptation, are consistent with the characteristics of the species known for its rusticity and adaptability. This study is the first exhaustive genome-wide analysis of the diversity of Italian donkey populations. The results emphasized the high informativeness of genome-wide markers retrieved through the ddRAD approach. The findings take on great significance in designing and implementing conservation strategies. Standardized genotype arrays for donkey species would make it possible to combine worldwide datasets to provide further insights into the evolution of the genomic structure and origin of this important genetic resource.

## Teaser

This is the first study deciphering the genetic structure of Italian donkey populations using SNP data. The results of this study confirmed the history of the investigated populations in terms of both genetic and geographical origins.

## Introduction

A widely accepted hypothesis proposes the origin of the domestic donkey (*Equus asinus*) in North Africa, in a region ranging from northern Sahara to Eritrea (Camac, 1986; Kimura et al., 2013; Mitchell, 2018). Studies on mitochondrial variability (Beja-Pereira et al., 2004; Kimura et al., 2011) and further paleontological hypotheses on the domestication origin outside the African continent (Todd et al., 2022) suggest that current domestic donkeys derive from two maternal lines. Those animals refer to the Nubian wild ass (*Equus africanus africanus*), and potentially to the Atlas wild ass (*Equus africanus atlanticus*) rather than to an undescribed subspecies that populated the Middle East (Cattani and Bökönyi, 2002).

Historically, donkeys have been widely used as animals of burden in several regions of all the continents and, in Europe, mainly in the Mediterranean area. With the advent of mechanization in agriculture and the progressive movement of people from the countryside to the cities, the number of donkeys has dramatically decreased leading to a general loss of genetic variability (Camillo et al., 2018; Carneiro et al., 2018; Seyiti and Kelimu, 2021). Furthermore, despite their economic importance, especially in developing countries, donkeys have received less attention than other livestock species. Progressively, a demographic decline of the *E. asinus* of up to 80% in the 20th century was observed, resulting in a dramatic loss of biodiversity and lack of information about populations and their uses in Europe (Camillo et al., 2018).

After a period of abandonment of many donkey populations, a demographic reversal has been noticed in developed countries (Rizzi et al., 2011). In particular, the growing interest toward donkey breeding is related to milk production due to its specific nutritional and nutraceutical value (Tidona et al., 2011; Brumini et al., 2015; Cavallarin et al., 2015; Li et al., 2018; Martini et al., 2018), its use in human health nutrition (Vincenzetti et al., 2017; Li et al., 2022), and its properties for cosmetics purposes (Kocic et al., 2020). Furthermore, thanks to their docility, donkeys are used for recreational and therapeutic purposes (Borioni et al., 2012; Galardi et al., 2022; Pécsek and Beke, 2023). As part of this productive differentiation, donkey breeding represents one of those livestock sectors that can promote the micro-economies of marginal areas thanks to its potential for local diversity conservation and territory exploitation, in line with the Common Agricultural Policy (Muscat et al., 2022).

Italy has traditionally been considered one of the cradles of European donkey breeding (Colli et al., 2013), and despite a considerable loss of biodiversity, today still counts nine autochthonous populations. The animals have undergone a substantial decrease in population size (77,269 heads registered in the national data bank in 2023; https://www.vetinfo.it/j6_statistiche/#/report-pbi/110, accessed on 9 February 2024) that might lead to high levels of inbreeding and potentially results in fitness depression, increasing the risk of breed extinction. In Italy, since 2022, the National Association of Equine and Donkey Breeders (A.N.A.R.E.A.I., accessed on 23 October 2023) has been managing the herd books of Equidae of limited diffusion, among which it is possible to find the Asinara, Pantesco, Viterbese, Romagnolo, Amiata, Sardo, and Ragusano breeds. The association A.N.A.M.F. (National Association of Murgese Horse and Martina Franca Donkey) holds the studbook of the Martina Franca breed (accessed on 23 October 2023). Grigio Siciliano is the only donkey population not officially recognized as a breed. The FAO agency attributes the Italian donkey breeds to a level of extinction risk ranging from vulnerable to endangered (Amiata, Sardo, Martina Franca, Ragusano, and Romagnolo) up to critical (Asinara, Pantesco, and Viterbese). Due to their very limited population size, some of these breeds are also included in conservation plans (e.g., Pantesco). Therefore, without immediate actions, the effective population size of these populations will be inadequate to prevent constant genetic loss in future generations (Bodò, 1992).

Over the last decades, microsatellite markers have been used to reveal genetic variability and the level of relatedness among some Italian donkey populations (e.g., Guastella et al., 2007; Bordonaro et al., 2012; Matassino et al., 2014). Analysis of genomic data is an invaluable tool for effective management of breeding programs, also in small populations, providing background information concerning genome structure in domestic animals (Mastrangelo et al., 2018b). The increasing use of high-throughput DNA analysis and genomic sequencing has enabled accurate assessment also of the donkey species, retrieving information concerning genome structure in different local populations (Renaud et al., 2018; Wang et al., 2020; Todd et al., 2022). Thus, the main objective of this study was to investigate the present-day genomic structure the nine Italian donkey populations and to provide information on their current conservation status. Due to the absence of a species-specific bead chip array, the double-digest restriction site-associated DNA (**ddRAD**) sequencing was used to overcome the limitations of previous studies based on microsatellite markers.

## Materials and Methods

All experimental procedures and sampling were approved by the Bioethics Committee of the University of Palermo: protocol code UNPA-CLE-98597. Blood samples were collected in compliance with the European rules (Council Regulation [EC] No. 1/2005 and Council Regulation [EC] No. 1099/2009) during routine health controls by the public veterinary service. The authors confirm that they have followed EU standards for the protection of animals used for scientific purposes.

### Sampling and DNA extraction

**Figure 1.**
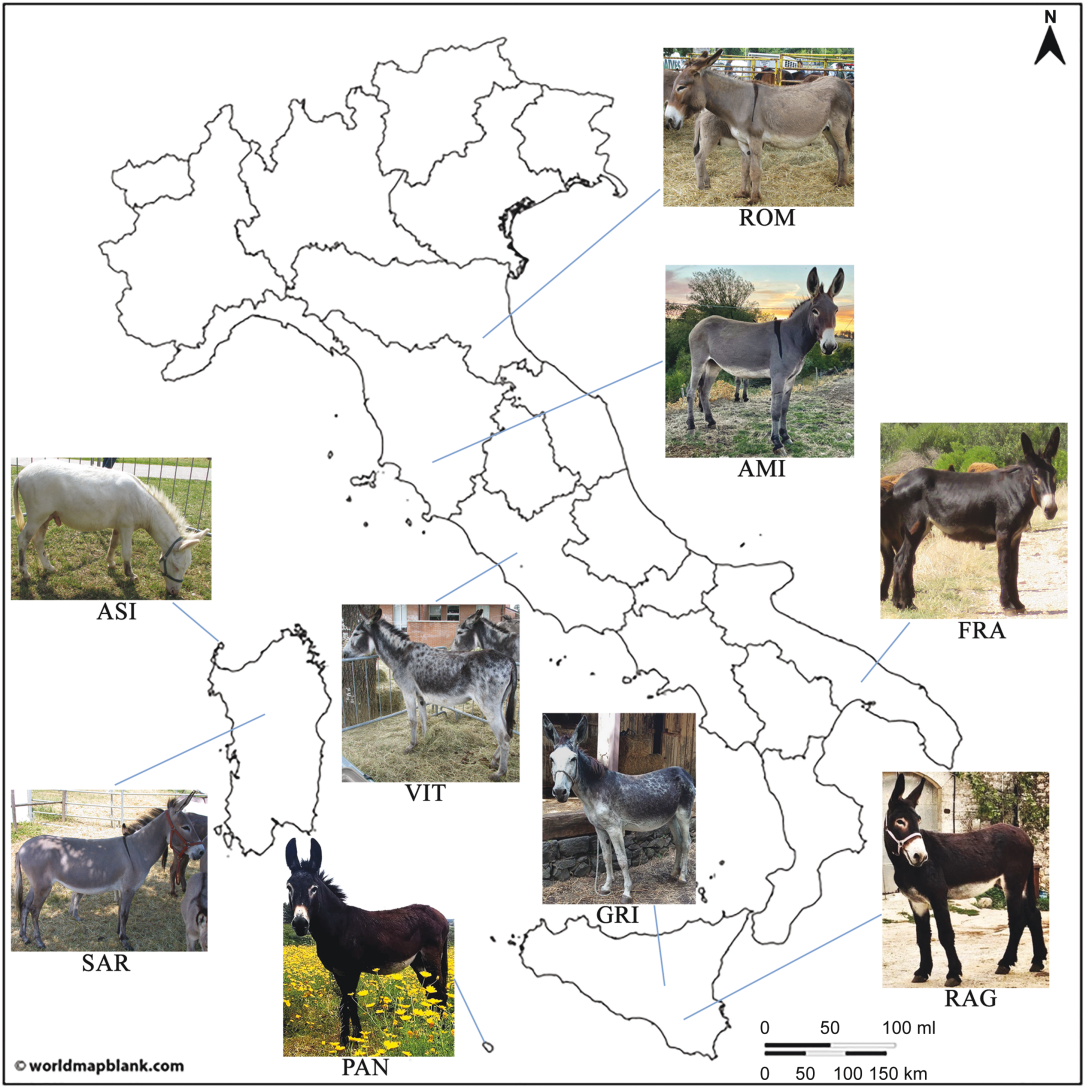
Geographic distribution of the nine investigated donkey populations in Italy.

Blood or nasal swab samples were collected from 220 individuals belonging to all nine Italian donkey populations, choosing animals that represent the latest generation. To the best of the authors’ knowledge, this dataset represents the largest and most complete one available for the donkey species in Italy and reflects its current genetic variability. Within each population, we selected unrelated or minimally related individuals sampled from different farms located in their traditional breeding areas (Figure 1). In detail, the populations involved in this study were Asinara (ASI = 18), Amiata (AMI = 22), Grigio Siciliano (GRI = 25), Martina Franca (FRA = 30), Pantesco (PAN = 23), Ragusano (RAG = 32), Romagnolo (ROM = 25), Sardo (SAR = 25), and Viterbese (VIT = 20). Genomic DNA was extracted from blood samples (using the commercial Illustra blood genomic Prep Mini Spin kit), or from nasal swabs (using MagMAX CORE Nucleic Acid Purification Kit). A short description of each population included in this study is reported in Supplementary Table S1.

### Library preparation and sequencing

Library preparation and sequencing were performed at IGA Technology Services (Udine, Italy), using a custom protocol after minor modifications to the original ddRAD protocol (Peterson et al., 2012). Genomic DNA was fluorometrically quantified, normalized to a uniform concentration and double-digested with the *Pst*I and EcoRI endonucleases. Fragmented DNA was purified by using AMPureXP beads (Agencourt) and ligated to barcoded adapters. Samples were pooled on multiplexing batches and bead-purified. For each pool, targeted fragments distribution was collected on BluePippin instrument (Sage Science Inc.). Each gel eluted fraction was amplified with indexed primers and subsequently bead-purified. The resulting libraries were checked both on a Qubit 2.0 Fluorometer (Invitrogen, Carlsbad, CA) and by a Bioanalyzer DNA assay (Agilent Technologies, Santa Clara, CA). Libraries were sequenced with 150 cycles in paired-end mode using NovaSeq 6000 instrument following the manufacturer’s instructions (Illumina, San Diego, CA).

### SNP call, filtering, and marker association

Initial raw data analysis as well as SNP calling was performed using IGA Technology Services in-house bioinformatics pipeline. Illumina reads were demultiplexed using the *process_radtags* utility included in the Stacks v2.61 (Catchen et al., 2013). BWA-MEM (Li and Durbin, 2009) with default parameters was used to align raw reads to the reference genome available on NCBI and released on 14 October 2021 (*E. asinus* ASM1607732v2, GenBank accession number GCA_016077325.2). Only uniquely aligned reads (i.e., reads with a mapping quality > 4) were used for downstream analyses. Detection of all the covered loci from the aligned reads was done using the *gstacks* program included in Stacks v2.61 (Catchen et al., 2013). The detected loci were filtered using the *populations* program included in Stacks v2.61 and run with the following options: –*R* = 0.75 in order to retain only the loci that were present in at least 75% of the whole metapopulation, and –*max*-*obs*-*het* = 0.8 in order to process a nucleotide site at a locus with an observed heterozygosity at maximum of 80%.

The retrieved raw data with 60,486 SNPs were filtered using the software PLINK ver. 1.9 (Chang et al., 2015) to exclude non-autosomal and unassigned markers, to remove animals with more than 10% missing genotypes, SNPs with a call rate lower than 90% and a minor allele frequency (**MAF**) lower than 1%. Due to high individual missingness, 23 samples were removed from the dataset.

### Genetic diversity indices

PLINK ver. 1.9 (Chang et al., 2015) was used to estimate within-populations genetic diversity coefficients, such as observed (*H*_O_) and expected heterozygosity (*H*_E_), molecular inbreeding coefficient (*F*_IS_), and the MAF. Historical effective population size (*h*Ne) was also calculated with the SNeP software using default settings (Barbato et al., 2015).

### Linkage disequilibrium analysis

The squared correlation coefficient of allele frequencies at pairs of loci (*r*^2^) was used as measure for the linkage disequilibrium (**LD**). The *r*^2^ value was estimated for all pairwise combinations of SNPs using Haploview software (Barrett et al., 2005). For each chromosome, pairwise *r*^2^ was calculated for SNPs between 0 and 1,000 kb apart (McKay et al., 2007). To visualize the LD pattern per population, *r*^2^ values were stacked and plotted as a function of inter-marker distance categories.

### Runs of homozygosity and islands investigation

Runs of homozygosity (**ROH**) were detected using PLINK ver. 1.9 (Chang et al., 2015) with the following parameters: 1) minimum of 1 Mb in length, 2) one missing SNP and not allowing heterozygous genotype, 3) the minimum number of SNPs for ROH was 20, 4) the minimum SNP density was set to one SNP per 100 kb, with a maximum gap length of 1,000 kb. The inbreeding coefficient (*F*_ROH_) per individual was calculated as follows:


$$\begin{array}{l} F_{ROH}=\frac{L_{ROH}}{L_{aut}} \end{array}$$

where *L*_ROH_ is the total length of ROH and *L*_aut_ is the length of the autosomal genome covered by SNPs (approximately 2,310 Mb). The shared genomic regions reporting ROH (ROH islands) were identified by calculating the percentage of SNPs present in a ROH based on the frequency of a SNP across all individuals. The top 0.995 SNPs of the percentile distribution were selected as threshold in the meta-population. Information on the annotated genes within the ROH islands was obtained from the Genome Data Viewer tool provided by NCBI (accessed on 18 September 2023) (Rangwala et al., 2021). Finally, we conducted a literature search to investigate the biological function of each annotated gene.

### Population structure analyses

To explore the genetic relationships among populations, the multidimensional scaling (**MDS**) analysis was performed based on pairwise identity-by-state (**IBS**) distances among individuals using PLINK ver. 1.9 (Chang et al., 2015). Moreover, Neighbor-Joining tree based on Allele Sharing Distances, calculated as one minus IBS, were visualized using SplitsTree4 ver. 4.14.8 (Huson and Bryant, 2006). Arlequin ver. 3.5.2.2 (Excoffier and Lischer, 2010) was used to estimate the between-populations variance using pairwise *F*_ST_, then visualized through a heatmap using the R package *ggplot2* (Wickham, 2016), and Reynolds’ pairwise genetic distances represented as a Neighbor-Joining tree using SplitsTree4 ver. 4.14.8 (Huson and Bryant, 2006). The analysis of genomic structure was performed by the software Admixture ver. 1.3.0 (Alexander et al., 2009) using the unsupervised model-based clustering algorithm from *K* = 2 to *K* = 15, which estimates the individual ancestry proportions given a *K* number of ancestral populations. The most likely number of clusters was estimated following the cross-validation procedure, whereby the estimated prediction errors are obtained for each *K* value. The estimated matrices were plotted through the R package BITE ver. 1.2.0008 (Milanesi et al., 2017).

Finally, to reconstruct the genomic relationships and migration events (*m*) among populations, a maximum-likelihood tree was built using the software TreeMix ver. 1.13 (Pickrell and Pritchard, 2012). A preliminary run was performed to check the optimal number of migration edges in 50 replicates ranging from 1 to 9, after assessing the best number of m using the *test.optM* function implemented in the R package OptM v0.1.6 (Fitak, 2021), 100 independent runs were performed at *m* = 2. We then compared tree likelihoods to build the consensus tree by retaining the tree(s) with the highest likelihood and unique topology. The analyses were performed with 2,000 bootstraps and considering the LD over blocks of 500 SNPs. The final consensus tree was visualized using the R package BITE ver. 1.2.0008 (Milanesi et al., 2017).

## Results

A total of 418,602,730 reads were generated and successfully demultiplexed to obtain a medium-density SNP genotypes panel. The average coverage per individual ranged from 2.51× to 59.10×, with a mean of 29.15×. After the stringent filtering for quality control, a total of 197 animals and 26,864 SNPs (distributed across the 30 autosomes) were retained for further analysis. The average number of SNPs per chromosome was 896, ranging from 345 (CHR18) to 2,836 (CHR02) (Supplementary Figure S1). The average distance between adjacent SNP pairs for the whole autosomal genome was about 85 kb.

### Genetic diversity indices

Genetic diversity indices of the nine donkey populations are reported in Table 1. *H*_O_ and *H*_E_ ranged from 0.179 ± 0.185 (ASI) to 0.241 ± 0.183 (RAG) and 0.198 ± 0.187 (ASI) to 0.240 ± 0.172 (RAG), respectively. ASI breed showed the lowest mean values also for MAF and *Ne*, whereas RAG had the highest. In agreement with this trend, the highest average *F*_IS_ was estimated in ASI, whereas RAG’s value was the lowest. As expected, estimated *Ne* decreased progressively through generations (from 761st to 13th; Supplementary Figure S2). Except for the GRI, RAG, and ROM, *Ne* was less than 90 for all donkey populations at 13th generation. The variation in *Ne* across generations was the smallest for ASI, FRA, and VIT. Ancestral populations of the contemporary AMI, GRI, RAG, and ROM exhibited the highest historical *Ne* values.

**Table 1.** Genetic diversity indices for the Italian donkey populations. Observed (*H*_O_) and expected (*H*_E_) heterozygosity, average MAF, inbreeding coefficient (*F*_IS_), inbreeding coefficient inferred from runs of homozygosity (*F*_ROH_) with related standard deviation values (s.d.) and effective population size relating to the 13th generation (*Ne*)

Breed	*H*_O_ ± s.d.	*H*_E_ ± s.d.	MAF ± s.d.	*F*_IS_ ± s.d.	*F*_ROH_ ± s.d.	*Ne*
AMI	0.234 ± 0.195	0.230 ± 0.175	0.164 ± 0.149	0.044 ± 0.184	0.044 ± 0.053	83
ASI	0.179 ± 0.185	0.198 ± 0.187	0.144 ± 0.154	0.273 ± 0.107	0.149 ± 0.100	47
FRA	0.215 ± 0.195	0.211 ± 0.183	0.152 ± 0.154	0.128 ± 0.054	0.092 ± 0.037	78
GRI	0.232 ± 0.176	0.237 ± 0.168	0.168 ± 0.144	0.060 ± 0.085	0.036 ± 0.057	111
PAN	0.222 ± 0.218	0.211 ± 0.196	0.158 ± 0.167	0.104 ± 0.085	0.054 ± 0.039	50
RAG	0.241 ± 0.183	0.240 ± 0.172	0.173 ± 0.151	0.021 ± 0.108	0.032 ± 0.030	127
ROM	0.239 ± 0.188	0.234 ± 0.172	0.166 ± 0.147	0.030 ± 0.047	0.013 ± 0.036	106
SAR	0.203 ± 0.180	0.216 ± 0.181	0.155 ± 0.151	0.176 ± 0.109	0.082 ± 0.078	83
VIT	0.222 ± 0.200	0.221 ± 0.177	0.158 ± 0.149	0.096 ± 0.060	0.015 ± 0.025	57

### LD analysis

Levels of pairwise LD decreased with increasing genomic distance between SNPs (Supplementary Figure S3). In general, the populations showed moderate LD decay, with the average *r*^2^ falling below 0.20 after 100 kb. Large differences across the Italian donkey populations were observed. The most persistent LD over distance was observed in the PAN and ASI breeds, with an average *r*^2^ > 0.1 for markers over 1,000 kb apart. Some populations (FRA, VIT, SAR, and AMI) showed intermediate LD decay. Average *r*^2^ *< *0.1 for markers over 100 kb apart was identified for GRI, RAG, and ROM.

### ROH and islands investigation

**Figure 2.**
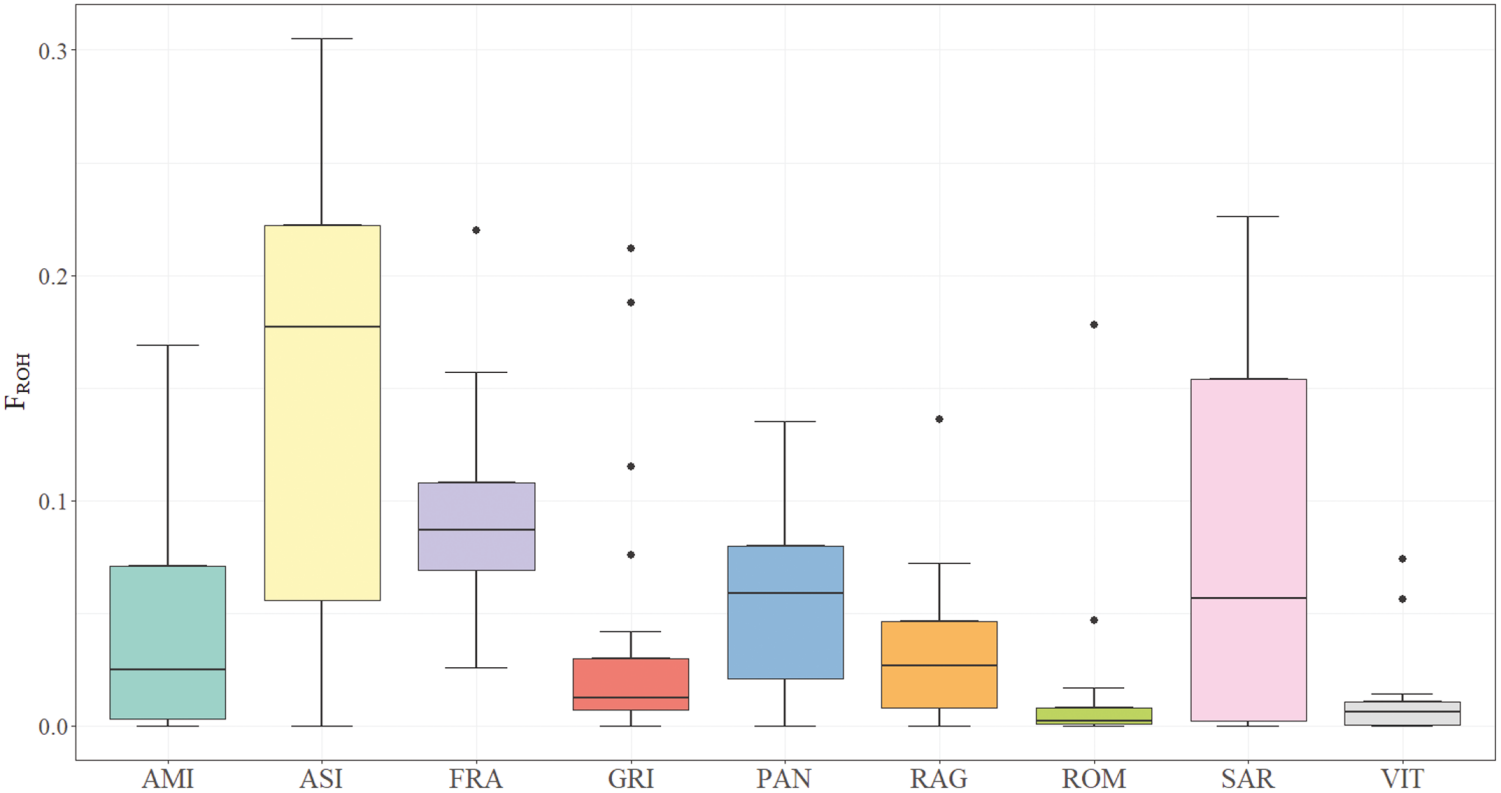
Box plot of the inbreeding coefficients inferred from runs of homozygosity (*F*_ROH_) for each donkey population.

Individual genomic inbreeding was also evaluated using the ROH approach. The distributions of the ROH inbreeding coefficients are shown in Figure 2 and the mean values are given in Table 1. The ROH coverage in the genome differed considerably among populations, with the highest mean values of *F*_ROH_ observed for the ASI and FRA breeds. In contrast, medium and low *F*_ROH_ were found for the other donkey populations that showed an average value below 0.09.

**Figure 3.**
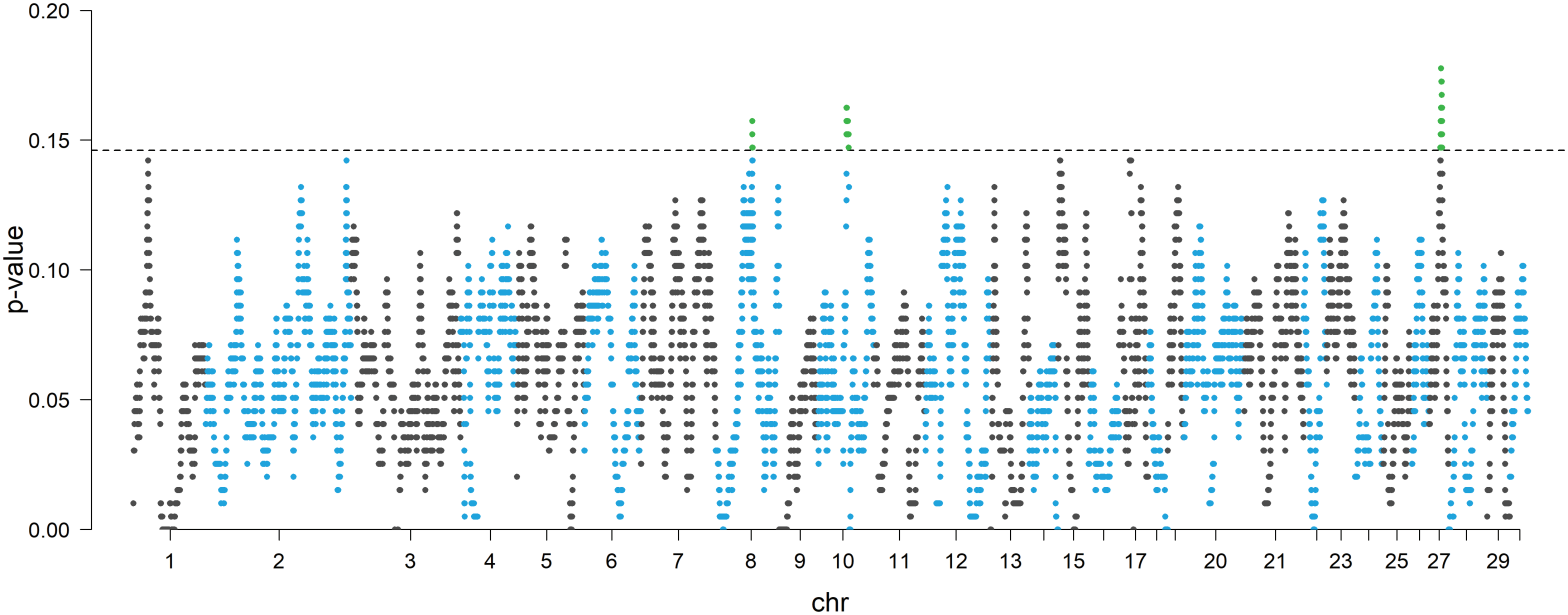
Manhattan plot of the incidence of each SNP in the ROH among all donkey populations.

The top 0.995% of the SNPs in the homozygosity range was considered to identify the genomic regions most associated with ROHs in the Italian donkey populations, reflecting a possible indicator of ROH hotspots in the genome (Figure 3). The chromosome position, number of SNPs, and start and end of these regions with the annotated genes are reported in Table 2. In total, we detected three ROH islands on CHR08, CHR10, and CHR27, ranging in length from 0.96 to 3.30 Mb. Within these ROH islands, we identified 83 known genes together with uncharacterized loci (**LOC**).

**Table 2.** ROH islands identified in Italian donkey populations. Chromosome number (CHR), start and end points (Start/End bp), number of SNP per region (n SNPs), length of region (in Mb), and genes inside the islands

CHR	Start bp	End bp	n SNP	Length (Mb)	Genes
8	59,766,272	60,723,588	41	0.96	*DDX39B, ATP6V1G2, NFKBIL1, LTA, TNF, LTB, LST1, NCR3, AIF1, PRRC2A, BAG6, APOM, C8H6ORF47, GPANK1, CSNK2B, LY6G5B, LY6G5C, ABHD16A, LY6G6D, LY6G6C, MPIG6B, DDAH2, CLIC1, MSH5, VWA7, SAPCD1, VARS1, LSM2, NEU1, SLC44A4, EHMT2, ZBTB12, C2, CFB, NELFE, SKIV2L, DXO, STK19, TNXB, ATF6B, FKBPL, PRRT1, AGPAT1, RNF5, AGER, PBX2, GPSM3, NOTCH4*
10	45,987,658	49,295,190	45	3.30	*UNC13B, FAM214B, STOML2, PIGO, FANCG, DNAJB5, PHF24, PGLYRP2, RASAL3, WIN, AKAP8L, AKAP8, BRD4, EPHX3, NOTCH, ILVBL, SYDE1, CASP14, CCDC105, SLC1A6*
27	18,523,665	21,668,292	67	3.14	*FRG1, ASAH1, PCM1, FGL1, MTUS1, PDGFRL, SLC7A2, MTMR7, VPS37A, CNOT7, ZDHHC2, MICU3, FGF20, MSR1, TUSC3*

### Population structure analyses

**Figure 4.**
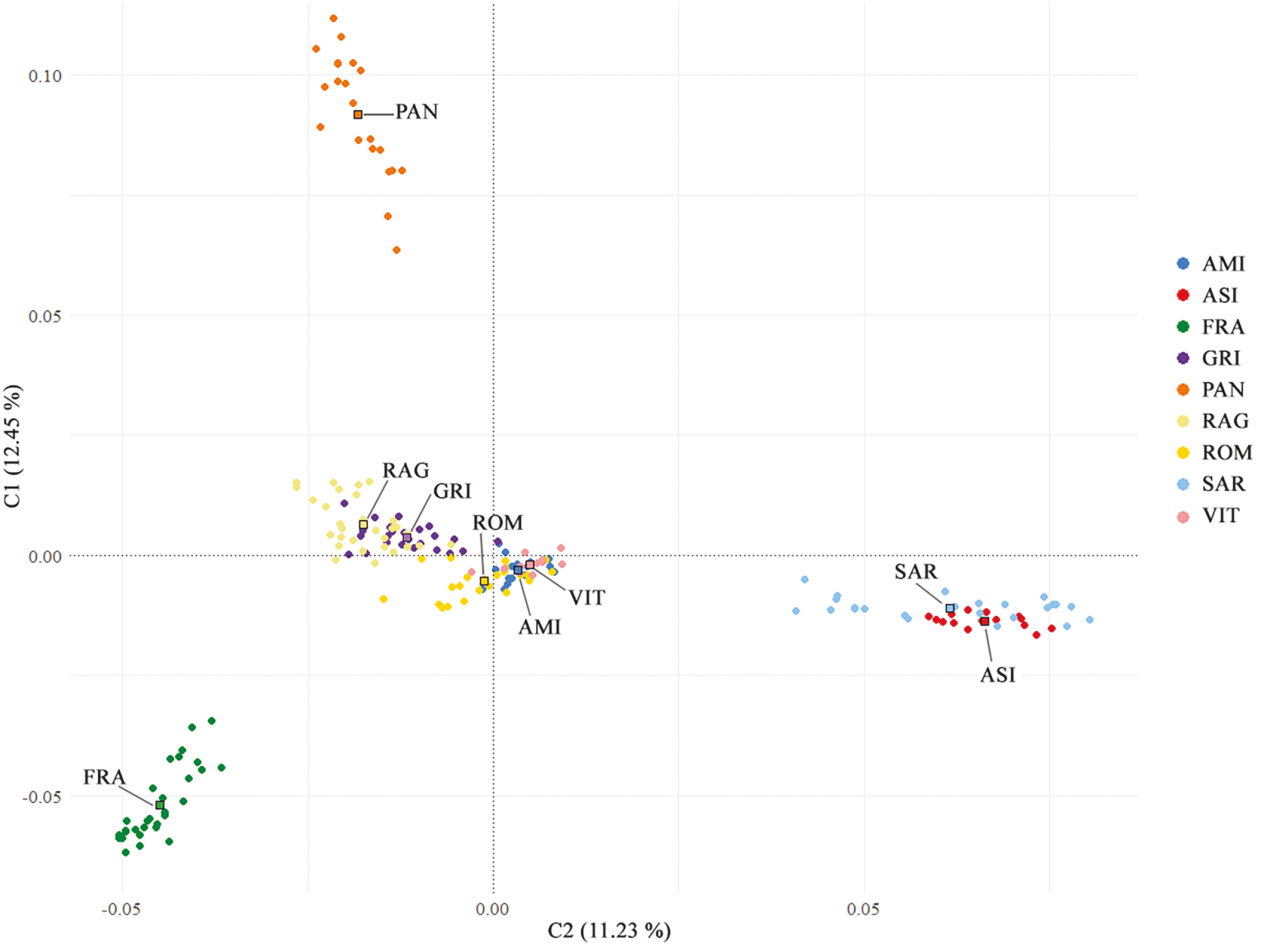
Multidimensional scaling (MDS) analysis of the nine donkey populations. The first (C1) and second (C2) components are presented in the x- and the y-axis, respectively.

We used a MDS plot of the pairwise IBS distances to identify the genetic relationship among the nine Italian donkey populations. The results showed a clear dispersion in the metric space of four main clusters (Figure 4). The first two components (C1 and C2) explained 23.68% of the total variance and separated PAN, FRA, and the two Sardinian breeds (ASI and SAR) which showed incomplete overlap. The rest of the donkey populations (RAG, GRI, ROM, AMI, and VIT) aggregated at the center of the plot with a partial admixture. In particular, RAG and GRI highlighted a high level of overlap, as did VIT, AMI, and ROM, forming a compact cluster. Focusing on the populations reared in Sicily, the results indicated that PAN was isolated from the other donkeys (RAG and GRI).

The pairwise fixation index (*F*_ST_) calculated among the nine donkey populations (Supplementary Figure S4) provided a representation of relationships that overlapped with that of the MDS. The results showed the lowest value between RAG and GRI (0.012) and the highest between PAN and ASI (0.174). Based on the results among all the populations, PAN was the most divergent. Consistent with the MDS plot, Neighbor-Net based on Reynold’ distances (Supplementary Figure S5) reported some clear clusters and relationships between populations. PAN, FRA, and Sardinian donkeys (ASI and SAR) showed a high divergence from the center of the tree. Within the Sicilian branch, RAG and GRI originated from the same branch and displayed a very close relationship. The shortest branch was observed for GRI, whereas the longest was found for PAN.

**Figure 5.**
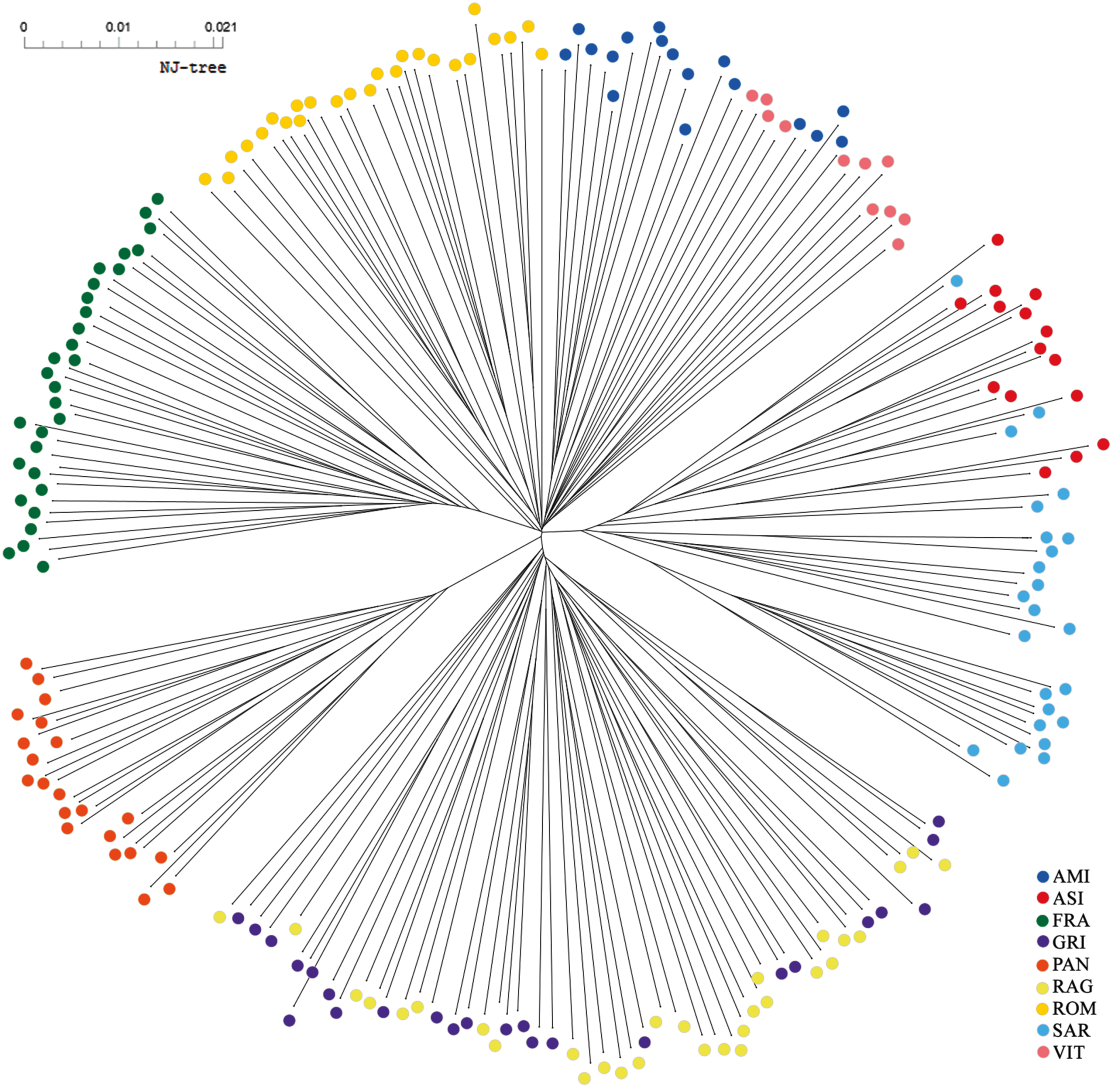
Neighbor-Joining tree based on Allele Sharing Distances among the individuals of the 9 Italian donkey populations.

The Neighbor-Joining tree based on allele sharing distance (1-IBS distances) gave a detailed picture of the relationships among all the individuals of the dataset (Figure 5). As already shown in the MDS plot, the populations with the highest differentiation were PAN and FRA. In fact, only for these populations, the results showed a perfect correspondence between individuals and the populations they belong to. The other donkeys reported a variable level of inter-population relationships. Specifically, all RAG and GRI individuals showed strong interconnection with each other. Within the cluster of donkeys from Sardinia, SAR highlighted a certain internal homogeneity interrupted by a few individuals mixed with ASI. Within the group of donkeys from Central Italy, AMI and VIT showed a reciprocal closer relationship than ROM.

**Figure 6.**
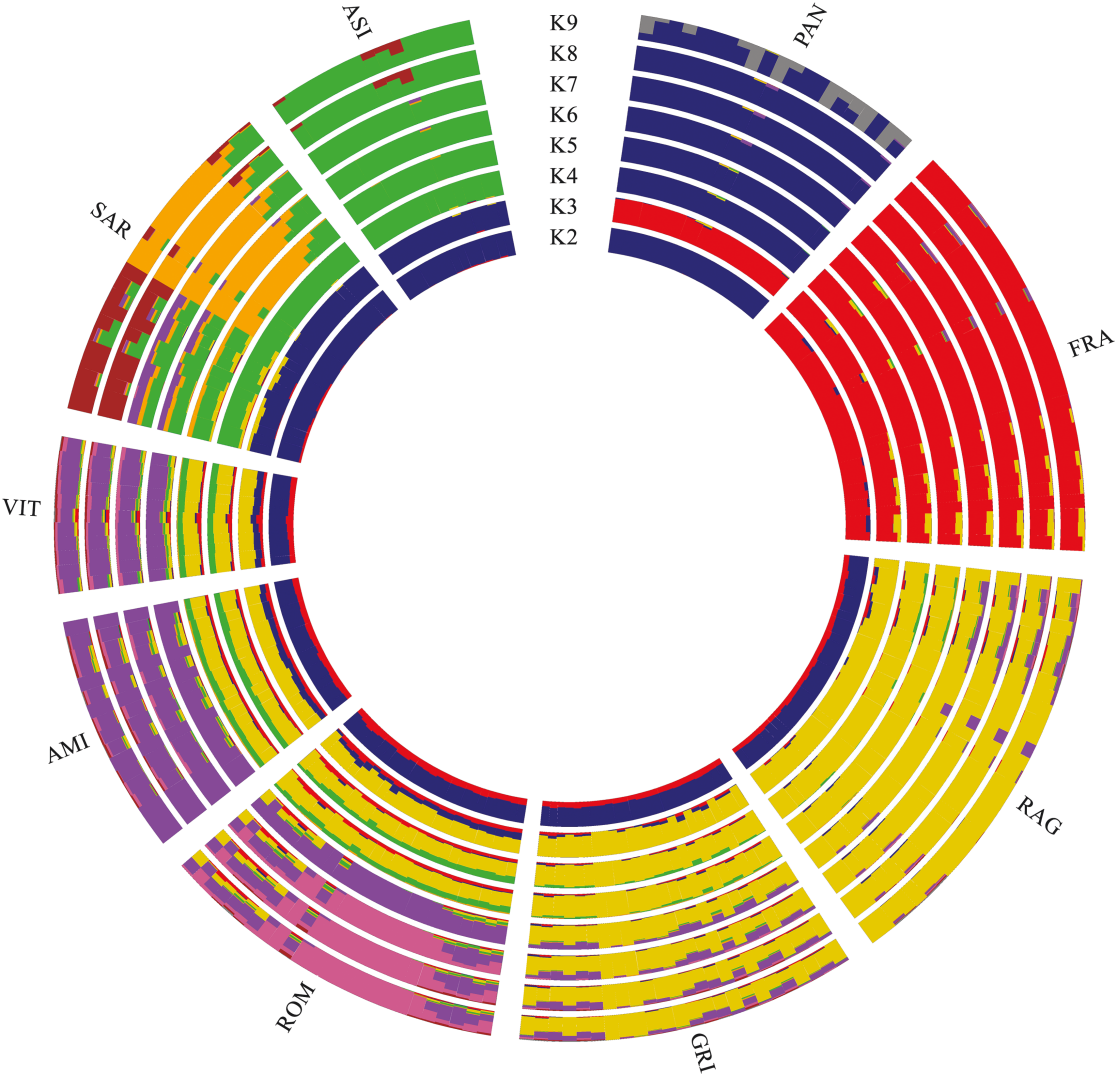
Circle plot of the ancestral clusters (K) inferred by the Admixture analysis of the 9 Italian donkey populations.

The results of the Admixture analysis are presented in Figure 6. The predictive likelihood value indicated *K* = 6 as the most probable number of clusters for the investigated populations (Supplementary Figure S6). At *K* = 2, FRA (red cluster) highlighted a clear separation, although a certain influence of this breed can be observed in all the other populations, except for PAN. At *K* = 3, the two Sardinian populations separated into a differentiated cluster (blue). Moreover, increasing the number of clusters, SAR showed an internal substructure with approximately half of genomic component attributable to ASI. The rest of the populations (RAG, GRI, ROM, AMI, and VIT), from *K* = 3 to *K* = 5, have shared genetic components predominantly referring to the gold cluster (Figure 6). At *K* = 6, the formation of the RAG–GRI (gold) vs. ROM–AMI–VIT (violet) clusters was highlighted. The range of clusters from *K* = 7 to *K* = 8 confirmed the previous groupings and highlighted the structuring of the ROM breed that differentiated from the AMI–VIT cluster. Finally, when the estimated clusters match the number of populations (*K* = 9), FRA and ASI confirmed their genomic distinctiveness, other populations showed internal substructure (SAR, ROM, PAN), and some populations highlighted shared genomic components (RAG–GRI and AMI–VIT).

**Figure 7.**
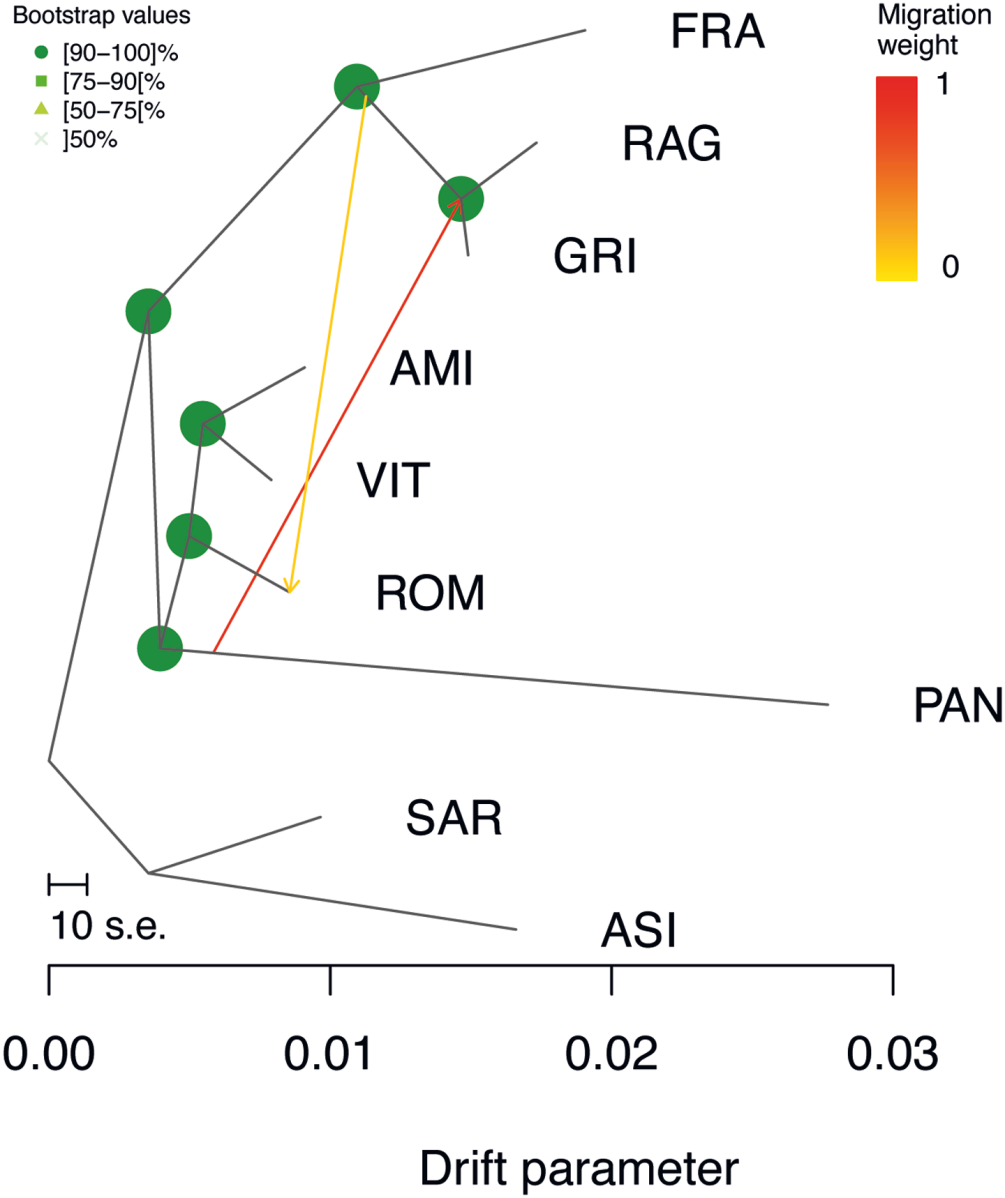
TREEMIX analysis with the most supported number of migration events (*m* = 2) for the nine donkey populations.

The Log-likelihood scores of the tested migration edges using the linear method as implemented in the *optM* function, indicated 2 as the most supported number of migrations. All the 100 independent runs showed a unique topology with a higher likelihood score of 347.5. Furthermore, all nodes were highly supported with bootstrap values higher than 90%. The graph showed a clear distribution of clusters according to geographic origin and highlighted ancestral relationships among donkey populations (Figure 7). At the basal position we found the two Sardinian breeds (SAR and ASI) which separated from all the rest, another group included donkey populations from Southern Italy and Sicily (FRA, RAG, and GRI) while the remaining group included Central Italy and the PAN breeds. However, it should be noted a very strong migration event from PAN to RAG and GRI, while a second weaker migration edge is observable from FRA to ROM.

## Discussion

In recent decades, populations of domestic or free-roaming donkeys have had mixed fortunes linked to alternative uses and conservation policies (Clancy et al., 2021). The little interest in this species has depleted a source of animal biodiversity that lends itself well to the use of marginal territories not suitable for agriculture or intensive breeding. The fundamental prerequisite for designing protection and recovery plans for animal genetic resources is the knowledge of their genomic structure and variability. Nowadays, SNP arrays are commonly used in genome-wide livestock studies thanks to the affordable cost, high genome coverage and consequent high level of information regarding the inheritance of genomic regions or biological processes (Baird et al., 2008; Gorjanc et al., 2015). However, a specific bead chip array is not available for all livestock species, such as donkey. The ddRAD sequencing represents a viable alternative to bead chip arrays and has attracted scientific interest due to the possibility of generating high-throughput polymorphism data with or without an existing reference assembly (Peterson et al., 2012; Maroso et al., 2018). Moreover, unlike fixed content platforms such as SNP arrays, ddRAD seq datasets are free of the ascertainment bias due to the non-representativeness of markers that can affect inferences of population studies (Mishra et al., 2020; Magris et al., 2022; Masharing et al., 2023), and can be applied to multiple studies, such as historical analyses (e.g., phylogenetic relationships, population structure) or functional processes surveys (Peterson et al., 2012). Genome wide SNPs based diversity and population structure analysis using ddRAD have been carried out in different livestock species such as cattle (Masharing et al., 2023), buffalo (Mishra et al., 2020), yak (Sivalingam et al., 2020), and dromedaries (Lado et al., 2020).

This study used the ddRAD analytics technique to characterize the pattern of genomic structure of nine donkey populations reared in Italy. This is the first genomic characterization of Italian donkey resources carried out using SNP markers, which provided insights into the genetic conservation status of the reared populations.

Our dataset revealed an average of demultiplexed reads per sample equal to 1.90 million of reads, similar to those reported in fish (2.36 million) (Magris et al., 2022), dromedary (1.93 million) (Lado et al., 2020), and cattle (2.20 million) (Masharing et al., 2023), but with a higher mean genome coverage of 29.15×, reflecting a higher accuracy in variant calling (Sims et al., 2014).

### Genetic diversity indices

Genetic diversity is a fundamental component of biological diversity, with great significance for population maintenance and adaptation to habitat changes. Improving our knowledge on the within-breed diversity and the between-breed relationships is fundamental for implementing conservation programs (Groeneveld et al., 2010).

The diversity estimates in the present study were lower than those reported in other studies on different donkey populations. For example, recently, Liu et al. (2023), using a medium panel of 45K SNP loci, showed higher values for MAF (0.250), *H*_O_ (0.347), and *H*_E_ (0.340) in Hetian Qing donkey, as well as Chen et al. (2023) in four Chinese donkey breeds. However, Lado et al. (2020), in a study on dromedary using ddRAD sequencing and 22K SNPs, showed mean values of genetic diversity indices like those estimated in the Italian donkey populations. In comparison with other livestock species, i.e., Italian goat (*H*_E_ = 0.35 to 0.41) (Nicoloso et al., 2015), sheep (*H*_E_ = 0.33 to 0.37) (Ciani et al., 2014), and cattle (*H*_E_ = 0.27 to 0.35) (Mastrangelo et al., 2018a), we consider the genome-wide diversity in Italian donkeys as moderate.

The MAF values were homogeneous among the populations, and, on average, SNPs were equally informative for all populations. For several populations the expected heterozygosity was slightly lower (AMI, FRA, PAN, and ROM) or close (RAG and VIT) to the observed heterozygosity, showing a moderate level of genetic diversity. For Asinara, the largest differences between *H*_O_ and *H*_E_ can be explained by a probable Wahlund effect because of the fragmentation of the breed into smaller groups corresponding to farms. This situation was also revealed in a previous study on this breed based on microsatellites (Colli et al., 2013).

Comparable genomic SNP data for these populations are currently not present in the scientific literature. In fact, to date, all studies on the genetic diversity of Italian donkey populations have been conducted using microsatellite markers (Bordonaro et al., 2012; Colli et al., 2013; Matassino et al., 2014). In a previous study, Colli et al. (2013), reported the Romagnolo as the most variable breed, with the highest *H*_E_ and the lowest *F*_IS_ values. In our study, the SNP data showed the Ragusano as the breed with the highest genetic diversity for both parameters. For *F*_IS_ value, our results were slightly different when compared with Colli et al. (2013); the authors reported the highest value for Sardo breed, whereas our data showed the highest value for Asinara followed by Sardo. Although a comparison among studies can be biased by the use of different marker sets and sampling, we highlighted an average inbreeding similar to the one reported in the previous research (Colli et al., 2013).

We observed a very small *Ne*, particularly for those populations with low numbers of breeding animals due to high inbreeding, bottlenecks, or geographical isolation, as in the case of Asinara, Pantesco, and Viterbese. In particular, the Asinara and Pantesco breeds had the lowest *Ne*, probably due to the small census and alternated period of isolated breeding that these breeds experienced since their creation, which is also consistent (particularly for Asinara) with their low genetic diversity. Moreover, for Pantesco, the results reflect the demographic history of the breed. Indeed, over the past thirty years, a genetic conservation program has been implemented for this breed, starting from nine donkeys (three males and six females) that were identified and used as founders to rebuild the breed (Bordonaro et al., 2012). We observed a decline in *Ne* with time in all donkey populations, as previously reported for other similar species as the horses (Capomaccio et al., 2023), indicating a consistent reduction in breeding animals in recent generations. The reduction, observed between about 60 to 13 generations ago, can represent the bottleneck these populations went into during the industrial era after the progressive reduction of their working role, as also shown for Italian horses (Capomaccio et al., 2023).

### ROH and homozygosity islands

We also used the well-established genomic tool of ROH to evaluate the impact of different management practices on Italian donkeys. Moreover, ROH analysis, providing the individual pattern of autozygosity within populations, constitutes an important tool for planning conservation schemes. Our results show some populations having a very low level of genomic inbreeding, as for example Romagnolo and Viterbese; most likely, these results can be explained by historical events, such as introgression. Animals showing high levels of *F*_ROH_, as observed in Asinara, Grigio, and Sardo, should be carefully used in breeding since they might suffer from inbreeding depression, and they likely be highly related to the rest of the population. Thus, highly related mating should be minimized to reduce the loss of genetic diversity and increase the biodiversity (Curik et al., 2014; Mastrangelo et al., 2018a). However, the *F*_ROH_ values observed for several populations (<0.05) suggest that the animals in this study are not highly inbred.

Within-population recurrence of ROH segments is widely used to identify genomic regions potentially under selection and involved in defining specific traits (Mastrangelo et al., 2017; Cesarani et al., 2018; Ablondi et al., 2019; Bordonaro et al., 2023). We explored the genomic regions associated with ROH in the meta-population, identifying shared common genomic regions in which reduced haplotype variability produces ROH islands (Cendron et al., 2021; Mastrangelo et al., 2021). In our study, we found three ROH islands with several known genes together with uncharacterized genes located on chromosome (LOC). Within the ROH island on CHR08, we found a particularly interesting group of genes (*DDX39B*, *ATP6V1G2*, *GPANK1*, *CSNK2B*, *LY6G5B*, *LY6G5C*, *ABHD16A*, and *LY6G6D*) associated with trypanotolerant response (Goyache et al., 2021). More in general, the three islands hosted several genes related with immune resistance: *PRRC2A*, *LY6G5B*, and *BAG6* (Petersen et al., 2021); *TNF, LTB, LTA, PRRC2A*, and *TNXB* (Carignano et al., 2018). Another gene, associated with somatic cell count and potentially conferring genetic resistance to mastitis (Chen et al., 2015) was *AKAP8* (CHR10). Several interesting genes linked to local adaptation were also identified. Chromosome 10 mapped the candidate gene *PIGO*, which is related mainly to metabolism indicators of maintaining genomic stability against UV radiation and molecular adaptation under hypoxia (Guo et al., 2021) and *DNAJB5* that encodes a member of the DNAJ heat shock protein 40 family of co-chaperone proteins (Lampis et al., 2018). This gene plays a vital role in the stress tolerance of immune cells, especially against heat stress, with molecular chaperone and anti-apoptosis effects in the maintenance of immune cell survival and internal stability (Vjestica et al., 2013); selection for this gene might be associated with heat tolerance in the donkey populations. Another gene on CHR10 is *BRD4*, involved with the climatic adaptation in wild boar (Chen et al., 2022). All these genes, related to immune response and local adaptation, are consistent with the characteristics of the species, known for their rusticity and adaptability.

### Genetic relationship and structure

The origins of the donkey species today reared in Italy are sometimes uncertain and linked to a distant past of domination and imports that probably begins with the Etruscans (2000 BC), who introduced donkeys from Africa into Spain and Italy (Kugler et al., 2008). The evolutionary history of current populations was strongly affected by mule production addressed to a now-distant use as a pack animal on farms and in military campaigns and influenced by the contingencies of geographical isolation. According to Mascheroni (1929), four breeds were historically recognized called Pugliese, Siciliano, Pantesco, and Sardo, which today are referred to Martina Franca, Grigio-Ragusano, Pantesco, and Sardo populations, respectively.

The genetic distances and relationships, as well as the population structure, were investigated through different approaches, and in general, the results largely agree with the breeding history of the donkey populations under investigation. Four main genetic strains, corresponding to Pantesco, Martina Franca, the donkeys reared in Sardinia (Asinara and Sardo) and a macro-cluster with the other five populations were highlighted by the MDS, Neighbor-Joining, ADMIXTURE, and TreeMix. The MDS (Figure 4) grossly separated the populations according to their genetic origin and/or to their geographical proximity between breeding areas. Colli et al. (2013), using microsatellite markers, highlighted the same relationships among these populations. Although the Italian donkey populations have different demographic histories, our results show that some, such as central-northern Italy and part of Sicilian populations, overlap in a cluster and cannot be easily discriminated; their MDS coordinates affected a narrow plot space due to a reduced within-population genetic variability. A similar pattern was described, using a medium-density SNP array, in Italian cattle (Mastrangelo et al., 2018a), with several breeds overlapping in a single macro-cluster. The distribution of the genetic diversity highlighted proximity-related patterns as pointed out by the low genetic differentiation (*F*_ST_) among local populations from the same geographic area, such as Grigio and Ragusano or among the populations of central-northern Italy. This genetic similarity can be explained not only by geographical proximity, but also by the management of the populations and the historical gene flow between them. For example, before being recognized as a breed in 1953, the Ragusano donkey constituted a single population with the Grigio (Mascheroni, 1929), in which both the bay coat (today of the Ragusano) and the gray one (typical of Grigio donkey) were admitted.

For breeds such as Pantesco and Martina Franca, all results emphasized their high differentiation. This result shows the success of proper management conservation plans, even for populations with close geographical origins or historical relationships, as was in the case of the Pantesco and the other Sicilian populations. The clear separation between the two main strains of Sicilian donkeys (Pantesco and Ragusano-Grigio) was also highlighted by Bordonaro et al. (2012) and Colli et al. (2013). The reduced variability of Pantesco can be explained by bottleneck due to restocking from a very limited number of founders. This breed went almost extinct during the 1980s, when the last purebred sire died, and was reconstituted thanks to a reduced number of individuals with about 80%–95% of Pantesco ancestry (Camillo et al., 2010), and probably with the presence of ancestral components from the other Sicilian populations. This would also explain the strong migration event depicted in TreeMix among these populations.

The Neighbor-Joining tree (Figure 5) based on individual 1-IBS distances gave a more detailed picture, in line with the evolutionary history of these genetic resources. The long branch observed for Pantesco might be attributed to a combination of small population size and relatively recent isolation. On the contrary, the divergence of Martina Franca is probably due to focused selection schemes of a population that, as the Ragusano donkey, counts primeval haplotypes and several Italian maternal lineages (Mazzatenta et al., 2021). Moreover, the phylogeny has highlighted not only the close relationship between Amiata, Viterbese, and Romagnolo but also the proximity to the basal node from which Martina Franca derives (Beretti et al., 2005). The Amiata donkey stood book reports the use of Romagnolo stallions during the last century (Bigi and Zanon, 2008). Maternal shared inheritance between Martina Franca and Romagnolo donkeys was previously shown (Cozzi et al., 2017), probably linked to the common origin of the two breeds deriving from the Pugliese ancestral strain (Mascheroni, 1929). The results of TreeMix (Figure 7) highlighted a migration event between these two breeds, that might reflect gene exchanges dating back to past events, supporting the hypothesis of historical gene flow. Martina Franca was the donkey most used to produce mules during the First World War and widely used during 1970 to improve body size and conformation of different Italian donkeys (Amiata, Romagnolo, and Ragusano) (Bigi and Zanon, 2008). No historical information was found for the Viterbese donkey, which possibly suffered from the genetic influence of Amiata and Romagnolo due to introgression from geographical proximity. Neighbor-Joining also corroborated the partial overlapping between Sardo and Asinara donkeys. Although characterized by different coat colors, Asinara and Sardo donkeys presented a certain degree of mixing, also reported by mtDNA shared inheritance (Cozzi et al., 2017). The reason is probably to be found in the management of these two populations, which at least in the not-too-distant past shared the same range (Pinna et al., 1993). Moreover, the Sardo divided into two different sub-populations, one of which could result from Asinara’s introgression, and the other (in orange in Figure 6) could represent its original strain. The Admixture analysis also corroborated all the results above reported and showed shared genomic components among several populations. The *K* = 6 plot showed several individuals sharing a substantial proportion of their genomic components among populations, such as those from central-northern Italy (Amiata, Romagnolo, and Viterbese) and Sicily (Ragusano and Grigio), resembling the Neighbor-Joining and well-representing the evolution of the genomic structure of the Italian donkey. This observation was also consistent with the TreeMix phylogram, which showed a similar distribution of the same clusters. The donkey populations that were the most homogeneous at lower *K* values also displayed the lowest heterozygosity level. On the other hand, for the populations displaying less distinct clusters at the best *K* value (6), such as Sardo, there was no evidence for high levels of inbreeding probably due to admixed origins, also suggesting the occurrence of crossbreeding. Martina Franca and Pantesco presented the lowest levels of admixture with other populations. This result was expected for Pantesco based on the historical information confirmed by the study book. In the case of Martina Franca, a different result was expected due to its introgression history into other populations to improve the morphological characteristics (Bigi and Zanon, 2008). Colli et al. (2013) identified *K* = 8 as the most probable number of ancestral groups, corresponding to the total number of populations analyzed. Notwithstanding similarities in the *K*s-based hierarchical genomic structure, the estimated admixture downstream of the characterization with ddRAD approaches showed slight divergences possibly associated to the evolutionary histories of the populations in the last decades and greater informativeness of genome-wide markers. Therefore, intentional (Martina Franca and Ragusano into the small-sized population) or occasional introgressions due to shared geographical areas (Sardo and Asinara), geographical isolation (Pantesco) and variable level of management of donkey populations might have determined the current degree of admixture (Bigi and Zanon, 2008; Bordonaro et al., 2012; Colli et al., 2013; Cozzi et al., 2017; Mazzatenta et al., 2021).

## Conclusions

The consistency of the results across different approaches agreed with the demographic history, the origin, and previous results on the nine donkey populations, suggesting that our conclusions are robust. The populations have preserved most of their distinctive characteristics, probably due to differences in genetic origin, environment, genetic isolation, and inbreeding. Both the differentiation level among populations and the genetic variability within populations are crucial factors supporting conservation plans. Genetic distances confirmed the history of these populations highlighting that, over the years, their genetic identity was maintained, and the genetic heritage remained preserved for most of the Italian donkey breeds.

The genetic diversity results presented here represent a starting point to exploit local donkey populations and can be crucial in outlining conservation strategies. Thus, efforts should be made to improve genetic diversity, to limit inbreeding levels and increase the populations’ size of this important reservoir of genetic diversity. It remains a matter of discussion what are the optimal weights to attribute to the components of genetic diversity between and within populations to define conservation schemes. These results highlighted the importance of using genomic information to reveal the genetic structure of each population and to provide an objective basis for decisions regarding the conservation of the Italian donkey populations. When standardized genotyping arrays will be adopted for this species, it will be possible to combine various datasets, including non-Italian donkey populations, in order to provide further insights regarding the evolution of the genomic structure and origin of these genetic resources and the prospects for valorization.

## Supplementary Material

skae165_suppl_Supplementary_Table_S1

skae165_suppl_Supplementary_Figure_S1

skae165_suppl_Supplementary_Figure_S2

skae165_suppl_Supplementary_Figure_S3

skae165_suppl_Supplementary_Figure_S4

skae165_suppl_Supplementary_Figure_S5

skae165_suppl_Supplementary_Figure_S6

## Data Availability

The datasets generated and/or analyzed during the current study are not publicly available but are available from the corresponding author upon reasonable request.

## Abbreviations

ddRAD: double-digest restriction site associated DNA
ASI: Asinara
AMI: Amiata
GRI: Grigio Siciliano
FRA: Martina Franca
PAN: Pantesco
RAG: Ragusano
ROM: Romagnolo
SAR: Sardo
VIT: Viterbese
*H*_O_: observed heterozygosity
*H*_E_: expected heterozygosity
*F*_IS_: molecular7 inbreeding coefficient
MAF: minor allele frequency
hNe: historical effective population size
LD: linkage disequilibrium
*F*_ROH_: inbreeding coefficient
*L*_ROH_: total length of ROH
*L*_aut_: length of autosomal genome covered by SNPs
MDS: multidimensional scaling
IBS: identity-by-state
ASD: allele sharing distances
*F*_ST_: fixation index
