# Supplementary material for: Analysis of ddRAD-seq data provides new insights into the genomic structure and patterns of diversity in Italian donkey populations
Source: J Anim Sci. 2024 Jun 14;102:skae165. doi: 10.1093/jas/skae165 (PMC11214105; doi:10.1093/jas/skae165)
Supplement: skae165_suppl_Supplementary_Table_S1 [file skae165_suppl_supplementary_table_s1.docx]

**Table S1.** Description of the nine Italian donkey populations

| **Amiata** | **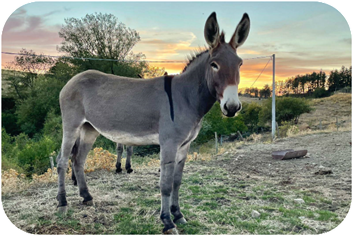** |
| --- | --- |
| This native breed of Tuscany region is currently used for trekking and as a mount, pet therapy and milk production. |  |
| It has a robust build, grey coat with dorsal stripe and scapular cross, light grey muzzle and belly. The height at the withers is on average 135 cm for males and 130 cm for females. |  |
| Registered animals: 2,623 (A.N.A.R.E.A.I.) |  |

| **Asinara** | **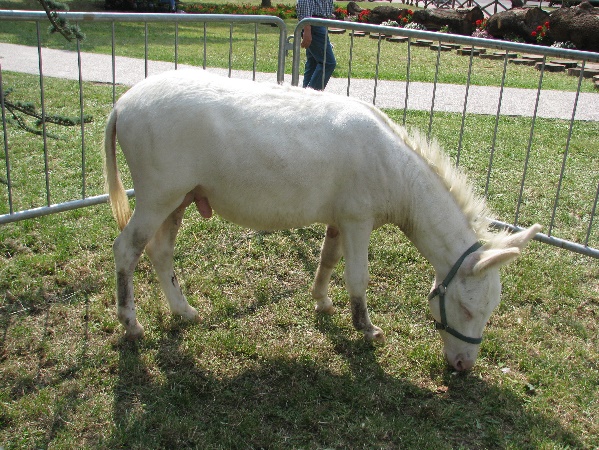** |
| --- | --- |
| Breed originating from the island of the same name (Asinara), northwest of Sardinia, where these feral donkeys live in wild conditions. |  |
| It has a small build, white coat, and pink skin. The height at the withers is on average 92 cm for both sexes. |  |
| Registered animals: 311 (A.N.A.R.E.A.I.) |  |

| **Grigio Siciliano** | **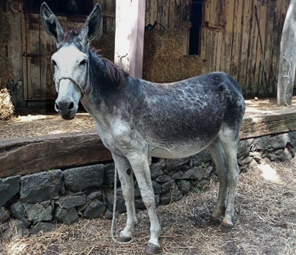** |
| --- | --- |
| This population native to the Sicily region, recently has been used for milk production in mixed farms with the Ragusano breed. |  |
| It has a robust constitution and a grey coat, both light and dark, sometimes with reddish highlights. The belly, the eye contour and the muzzle are white. The height at the withers is on average 124 cm for females. |  |
| Estimated consistency: 100 subjects |  |

| **Martina Franca** | 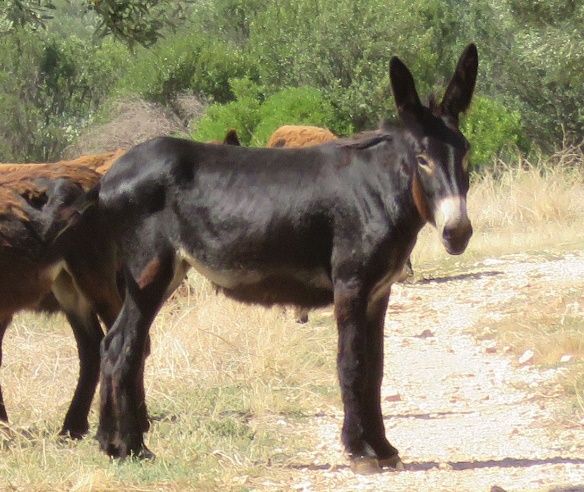 |
| --- | --- |
| Breed originally from Puglia. This donkey is employed mainly for milk production and with a recent slight interest in mule production. |  |
| It has a robust build, a black coat, with belly, inner thighs and muzzle of white-grey color. The height at the withers is on average 135 cm for males and 127 cm for females. |  |
| Registered animals: 850 (A.N.A.M.F.) |  |

| **Pantesco** | **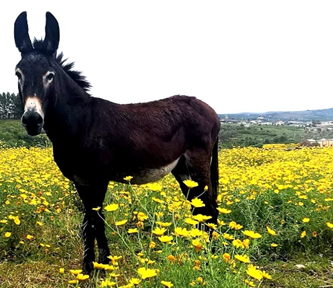** |
| --- | --- |
| Breed originating from the island of Pantelleria, southwest of Sicily. The current breed recovery program involves the production of mules intended for work within nature reserves. |  |
| It has a dolichomorphic constitution, black or dark bay coat, and belly, inner thighs, and muzzle of white color. The height at the withers is on average 132 cm for both sexes. |  |
| Registered animals: 85 (A.N.A.R.E.A.I.) |  |

| **Ragusano** | **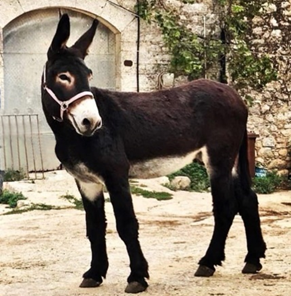** |
| --- | --- |
| This autochthonous breed from the Sicily region is currently employed for milk production, trekking, and riding. |  |
| It has a robust constitution, dark bay coat with a light belly and inner thighs tending towards white; muzzle and eye contour of grey color. The height at the withers is on average 138 cm for males and 130 cm for females. |  |
| Registered animals: 3,712 (A.N.A.R.E.A.I.) |  |

| **Romagnolo** | **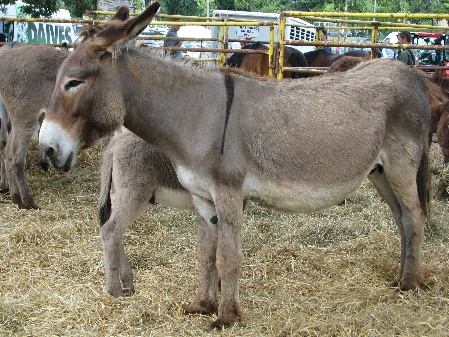** |
| --- | --- |
| This breed originates from the Emilia-Romagna region, it is currently employed mainly for milk production and also for trekking and pet therapy. |  |
| It has a robust constitution. The most diffused coat color is grey with a dorsal stripe, scapular cross and stripes on the forelimbs; the belly and medial face of the limbs are of light color. The height at the withers is on average 145 cm for males and 137 cm for females. |  |
| Registered animals: 995 (A.N.A.R.E.A.I.) |  |

| **Sardo** | **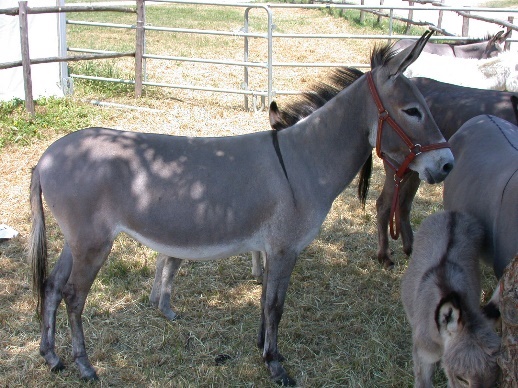** |
| --- | --- |
| This breed is originally from the Sardinia region and has been recently employed for trekking activities. |  |
| It has a small build, a grey coat with a dorsal stripe and a scapular cross; the belly and the inner face of the limbs have light hair. The height at the withers is on average 97 cm for both sexes. |  |
| Registered animals: 2,358 (A.N.A.R.E.A.I.) |  |

| **Viterbese** | **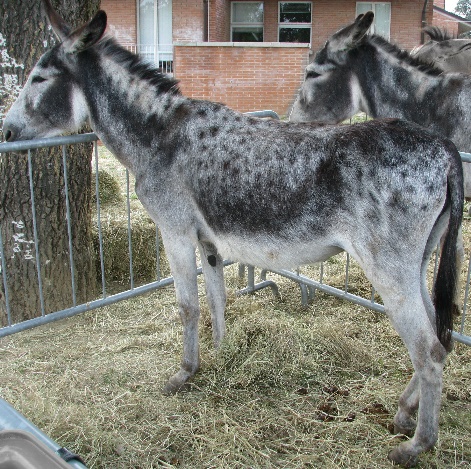** |
| --- | --- |
| This Native breed from the Lazio region is currently employed for equestrian competitions, pet therapy and milk production. |  |
| It has a robust constitution, with a brownish-grey to light grey coat with a dorsal stripe; belly, limbs and muzzle are of light grey color. The height at the withers is on average 128 cm for males and 123 cm for females. |  |
| Registered animals: 265 (A.N.A.R.E.A.I.) |  |
