# Supplementary figures and images for: Analysis of ddRAD-seq data provides new insights into the genomic structure and patterns of diversity in Italian donkey populations
Source: J Anim Sci. 2024 Jun 14;102:skae165. doi: 10.1093/jas/skae165 (PMC11214105; doi:10.1093/jas/skae165)

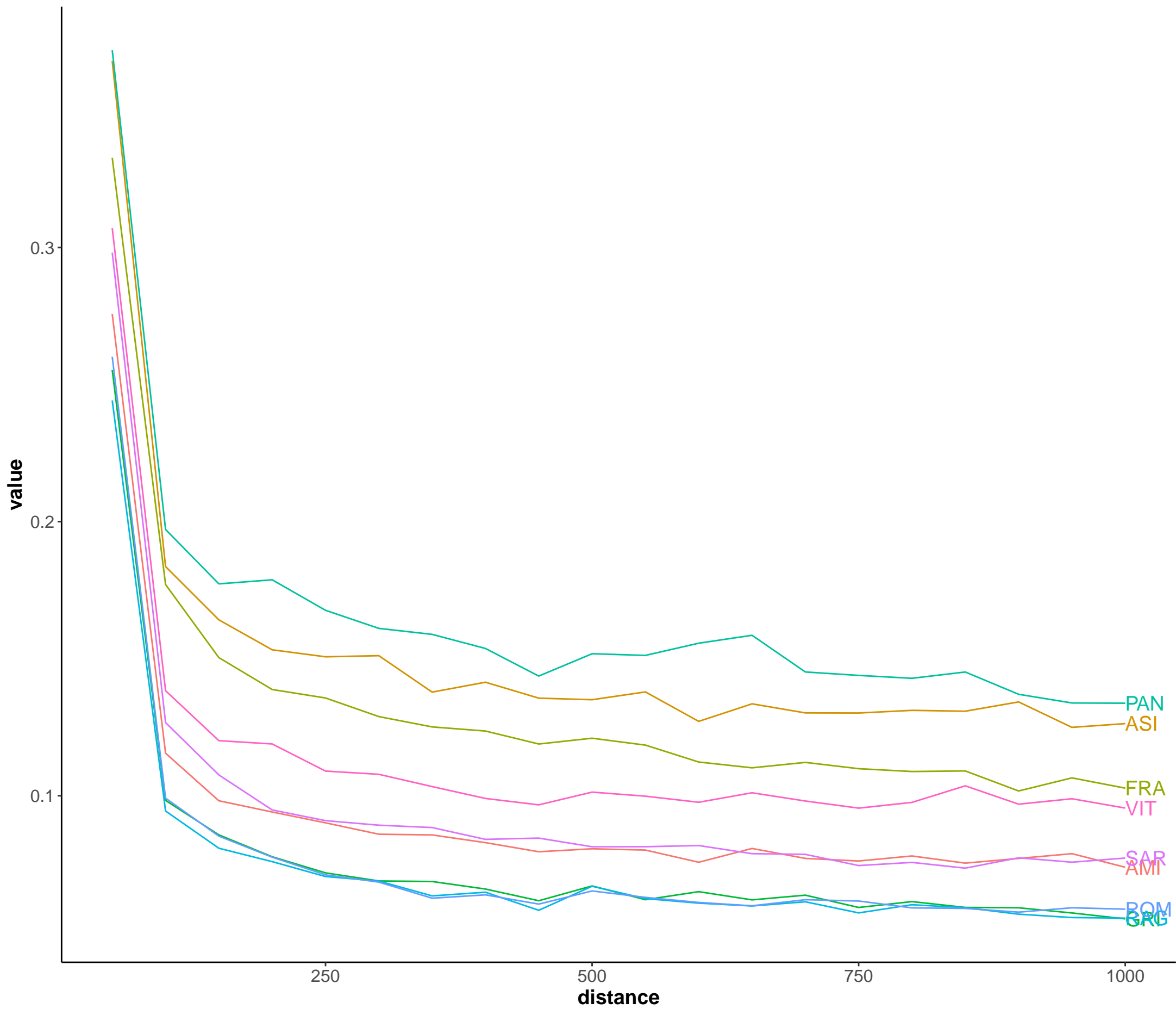

Supplement: skae165_suppl_Supplementary_Figure_S1 [file skae165_suppl_supplementary_figure_s1.pdf]

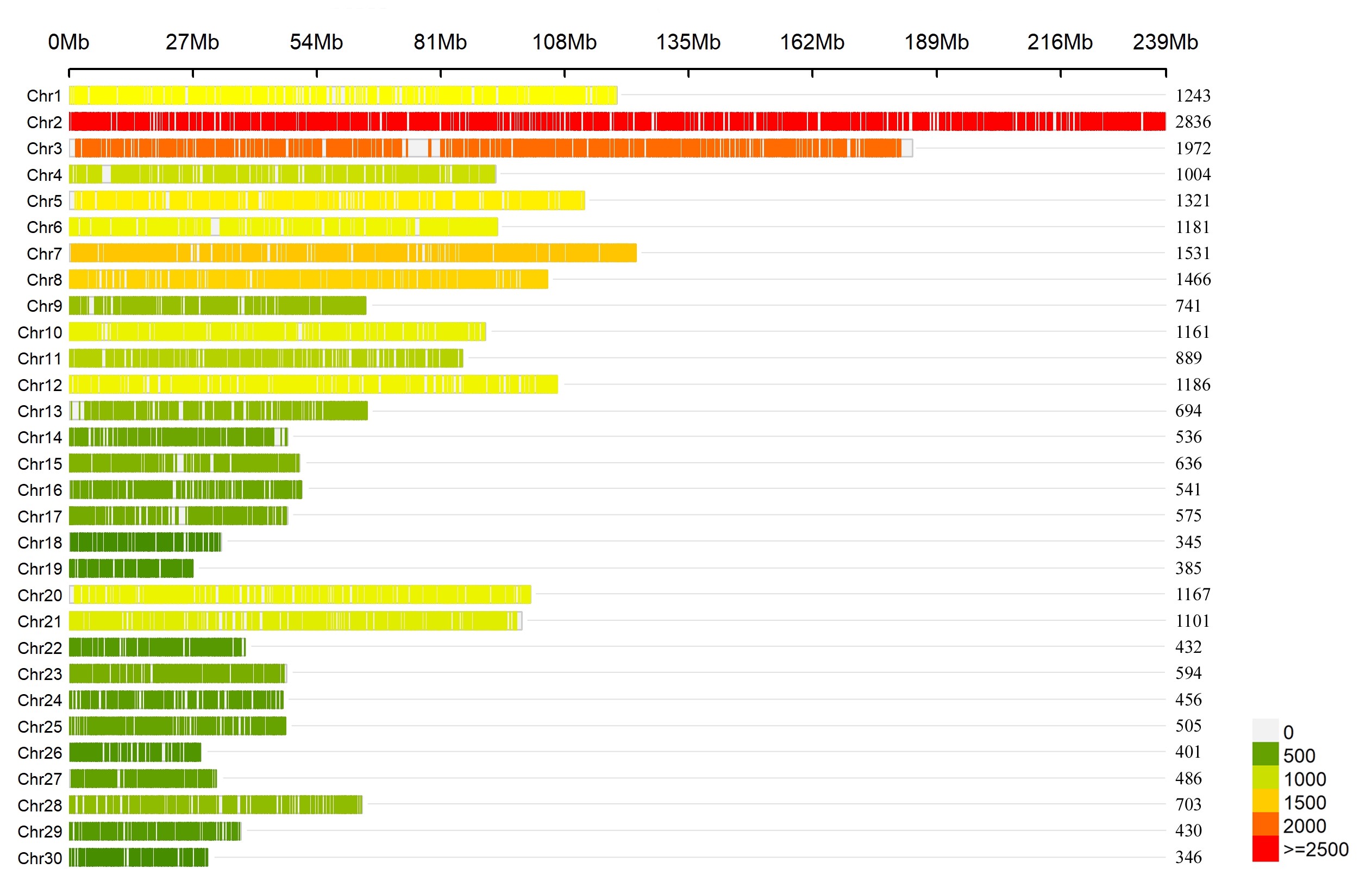

Supplement: skae165_suppl_Supplementary_Figure_S2 [file skae165_suppl_supplementary_figure_s2.jpeg]

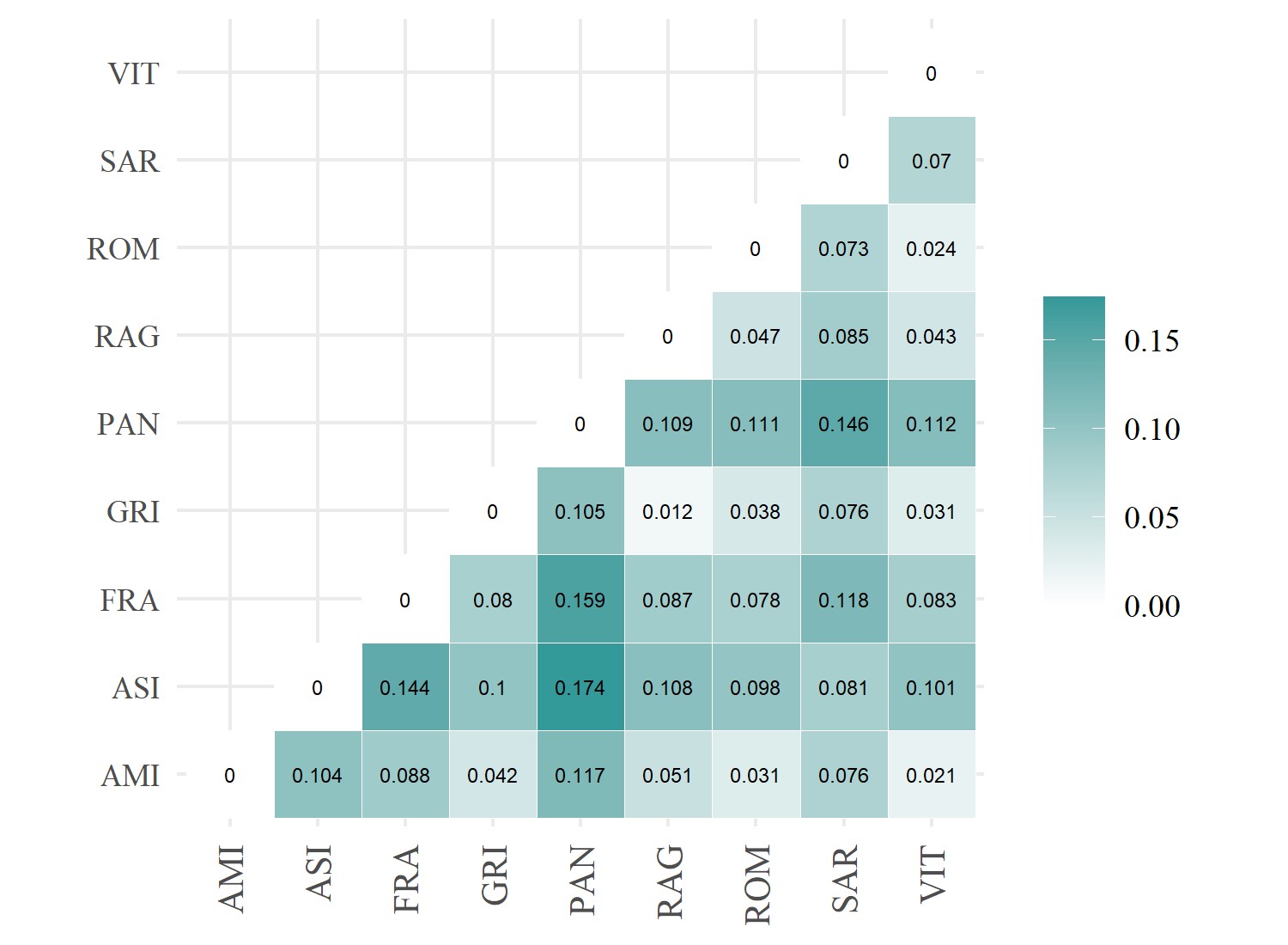

Supplement: skae165_suppl_Supplementary_Figure_S3 [file skae165_suppl_supplementary_figure_s3.jpeg]

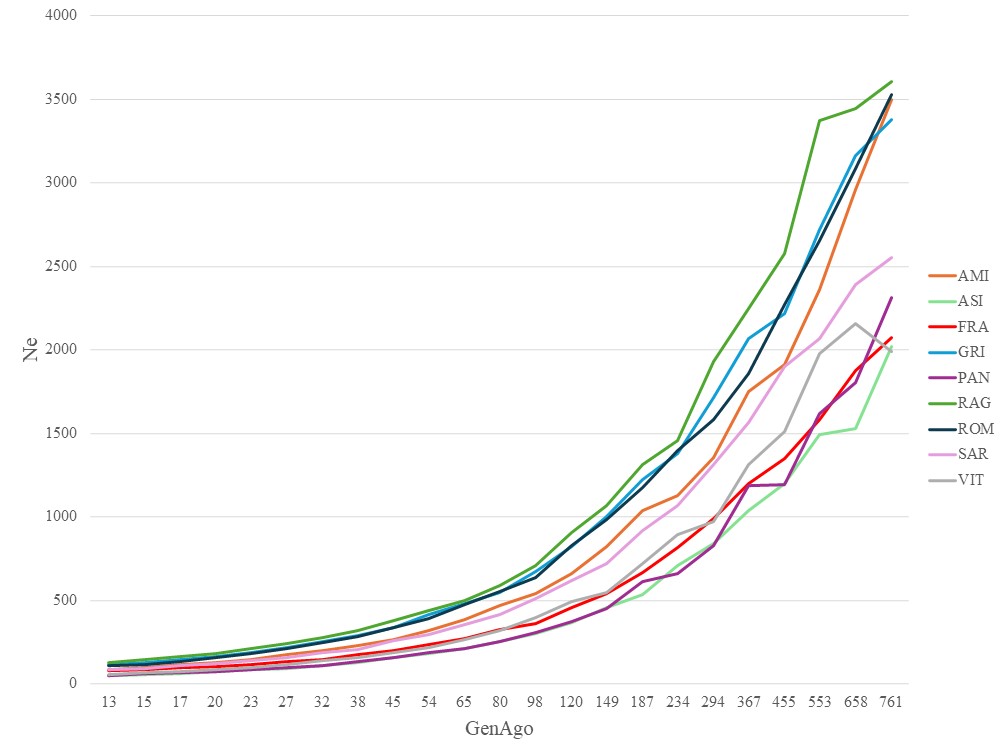

Supplement: skae165_suppl_Supplementary_Figure_S4 [file skae165_suppl_supplementary_figure_s4.jpeg]

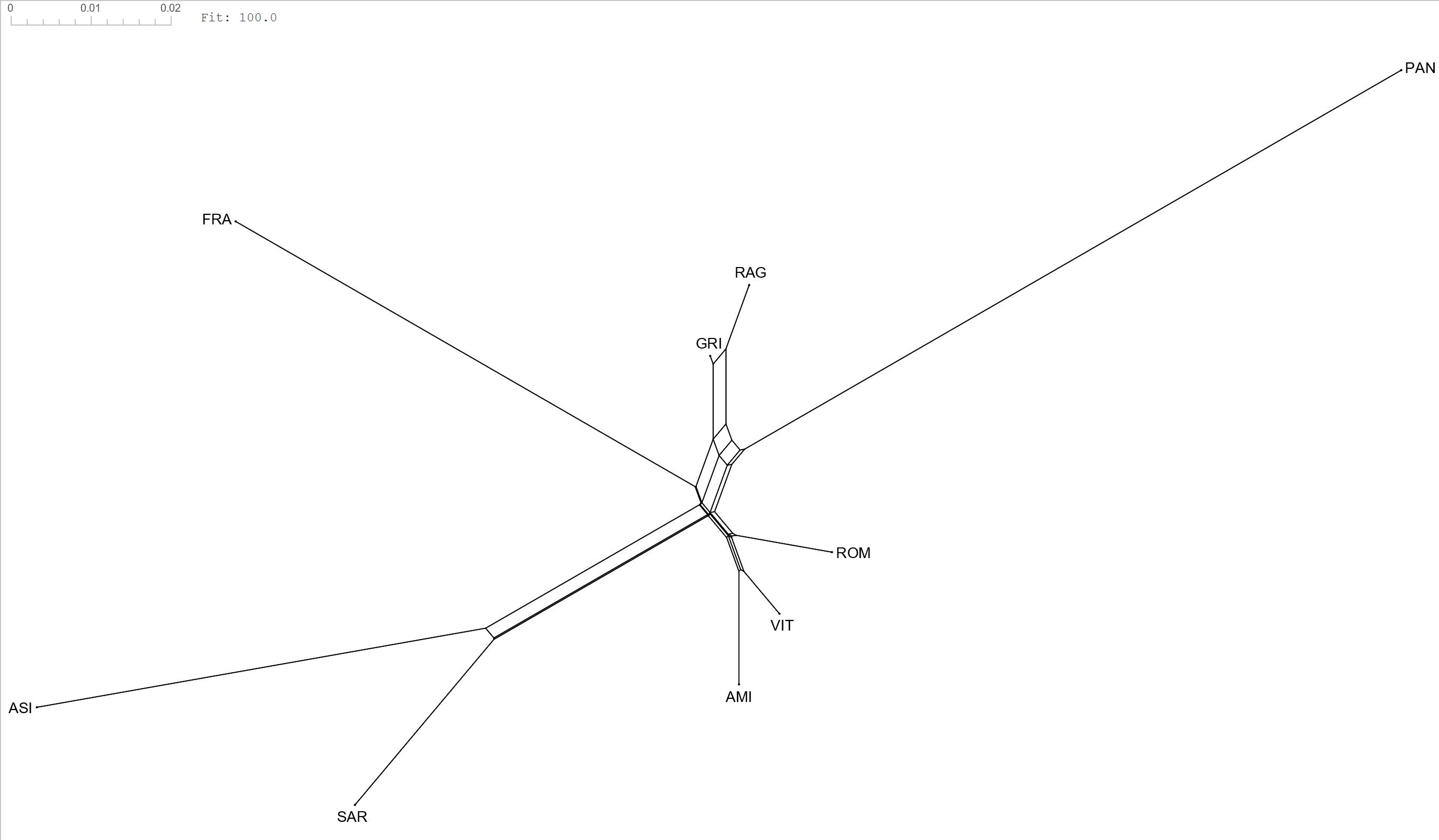

Supplement: skae165_suppl_Supplementary_Figure_S5 [file skae165_suppl_supplementary_figure_s5.jpeg]

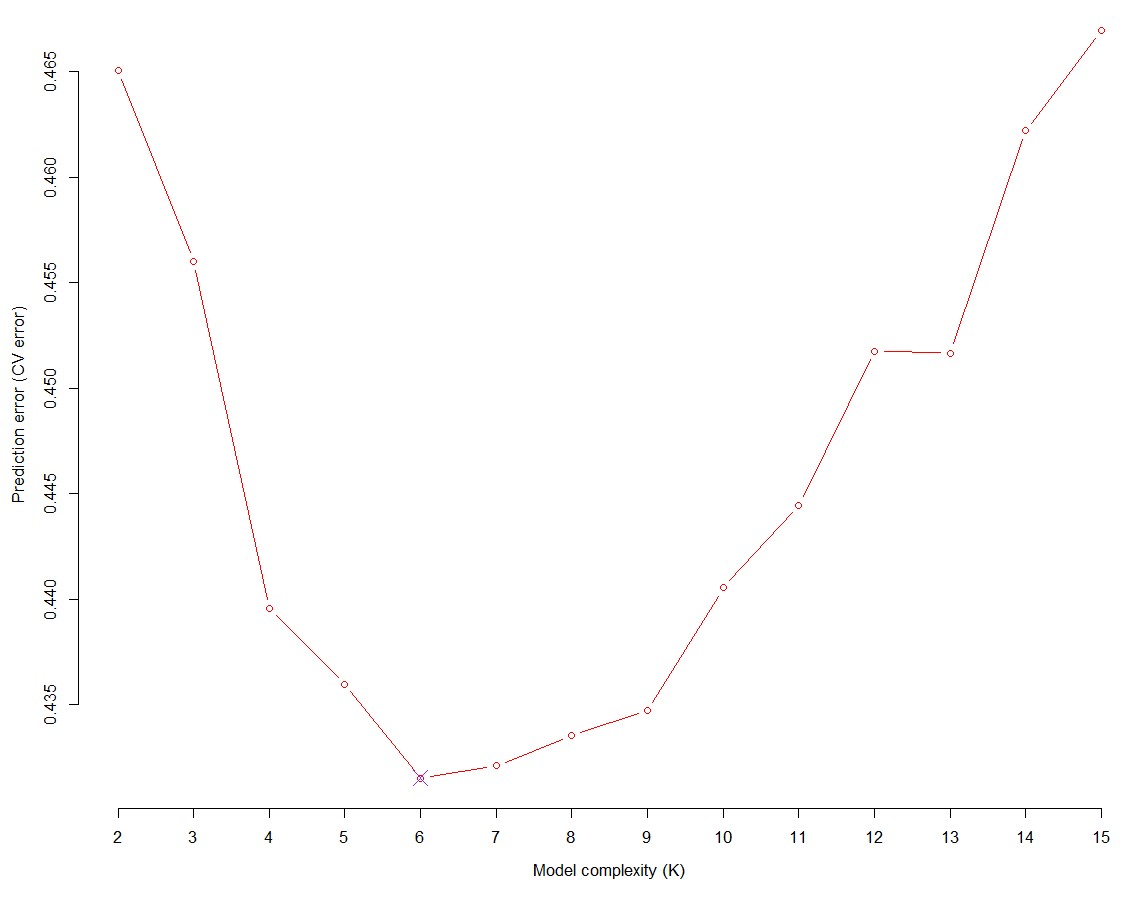

Supplement: skae165_suppl_Supplementary_Figure_S6 [file skae165_suppl_supplementary_figure_s6.jpeg]
